# Face2face: advancing the science of social interaction

**DOI:** 10.1098/rstb.2021.0470

**Published:** 2023-04-24

**Authors:** Antonia F de C. Hamilton, Judith Holler

**Affiliations:** ^1^ Institute of Cognitive Neuroscience, University College London, London WC1N 3AZ, UK; ^2^ Donders Institute for Brain, Cognition & Behaviour, Radboud University, 6525 GD Nijmegen, The Netherlands; ^3^ Max Planck Institute for Psycholinguistics, 6525XD Nijmegen, The Netherlands

**Keywords:** cognition, social interaction, neuroscience, communication, visual signals

## Abstract

Face-to-face interaction is core to human sociality and its evolution, and provides the environment in which most of human communication occurs. Research into the full complexities that define face-to-face interaction requires a multi-disciplinary, multi-level approach, illuminating from different perspectives how we and other species interact. This special issue showcases a wide range of approaches, bringing together detailed studies of naturalistic social-interactional behaviour with larger scale analyses for generalization, and investigations of socially contextualized cognitive and neural processes that underpin the behaviour we observe. We suggest that this integrative approach will allow us to propel forwards the science of face-to-face interaction by leading us to new paradigms and novel, more ecologically grounded and comprehensive insights into how we interact with one another and with artificial agents, how differences in psychological profiles might affect interaction, and how the capacity to socially interact develops and has evolved in the human and other species. This theme issue makes a first step into this direction, with the aim to break down disciplinary boundaries and emphasizing the value of illuminating the many facets of face-to-face interaction.

This article is part of a discussion meeting issue ‘Face2face: advancing the science of social interaction’.

## 1. Introduction

The natural ecological niche for some of the most fundamental human social interactions—conversations, confrontations and the bonding between parent and infant—is face-to-face. Finding a scientific understanding of the processes which are at work in a face-to-face social interaction is both important and challenging. It is important because it will allow us to understand some of the basic behavioural, cognitive and neurocognitive systems that make us human, and their evolution, to gain new insights into psychiatric disorders which are diagnosed and manifest in atypical interpersonal behaviours (e.g. autism and schizophrenia) and to create the next generation of artificial agents which can communicate with people (going beyond Amazon Alexa). However, face-to-face interaction is challenging to study because natural human (or animal) behaviour is hard to pin down in the laboratory and manipulate experimentally.

In this special issue, we bring together papers from a wide variety of approaches to the study of face-to-face interaction, from animal behaviour to linguistics and from computational models to infant development. The papers included here showcase new types of experimental designs and paradigms, including novel technologies to capture data and create stimuli, and they break new ground in terms of analytic methods. These techniques can be applied to typical adults, to child development, to atypical populations and to non-human species in order to understand the core principles of face-to-face interaction and their diversity in different settings. In this introductory paper, we will set out the principles of why face-to-face interaction matters and the overall frameworks within which we can take a scientific approach to this complex topic.

## 2. Studying face-to-face interaction

In addition to understanding face-to-face interaction being important because it is a fundamental human capacity, one of the strongest arguments for studying it directly comes from findings which show that behaviour, brain processes and cognition operate differently when people are in an interaction with another person. An increasing number of studies show that people perform differently when being watched by a real person [1–3], when socially co-present [4] and engaged in joint action [5] or taking part in a dynamic conversation [6–8]. While studying social interaction has been the very focus of enquiry in conversation analysis since its emergence in the 1970s [9–11], results like these have led to the importance of ecological validity and natural behaviour increasingly being recognized also in the cognitive and neurosciences [12,13]. This is expressed as an increased focus on second-person neuroscience [14].

However, there are also substantial challenges involved in building an experimentally grounded science of face-to-face interaction. We must move beyond stimuli reduced to individual words or individual faces, heard or seen in isolation and out of context, to studying real people or animals showing natural behaviours in interaction, including the deeply multi-modal nature of face-to-face interaction [15–17] and their core role in coordinating it [18–21]. This requires new types of experimental designs and paradigms, new technologies to capture data and create stimuli, and new analytic methods, all of which are now emerging. For example, automated video analyses and motion capture devices allow us to analyse large datasets and track faces and bodies in exquisite detail while machine learning lets us comprehend the resultant data. Wearable brain imaging systems such as functional near-infrared spectroscopy (fNIRS) permit tracking of brain activity patterns during conversation while virtual agents let us manipulate the details of social interaction in a controlled fashion. All these techniques can be applied to typical adults, to child development, to atypical populations and to non-human species in order to understand the behavioural, cognitive and neural mechanisms underpinning the core principles of face-to-face interaction and their diversity in different settings.

This special issue brings to the foreground the extremely rich, complex and multi-layered nature of human face-to-face interaction, and the inability to answer the many questions it gives rise to from a single disciplinary perspective. To understand face-to-face interaction in its entirety, studies must shed light on the many different elements and processes that play into it. The aim of this special issue thus is to emphasize the strong need for an interdisciplinary, multi-method and multi-level approach to understanding face-to-face interaction. This includes studies that focus on behaviour, cognition or neural mechanisms, studies that focus on words or vocalizations as means of coordination, others that zoom in on visual signals or use multi-modal approaches, studies that focus on animals, artificial agents or humans, and within the latter group there are studies on different age groups or populations with diverse traits and dispositions. Moreover, we need both, studies that focus on smaller samples for micro-analyses of behaviour that can shed light on the intricate behavioural processes that govern interactions, but which may not provide opportunities for generalization, as well as studies which do so based on larger samples, but which tend to gloss over the details of interaction [22]. Thus, we need brain and behaviour, micro- and macro-perspectives, automated and manual methods, since *together* they provide us with the rich array of opportunities necessary to gain comprehensive insight into the complex explanandum that is ‘face-to-face interaction’.

Critical to such a multi-disciplinary endeavour is mutual appreciation. Appreciation for what each discipline and type of approach can bring to the table in its own way and the contribution it offers in its own right, but also to go beyond parallel existence to seeing opportunities for integration and possibilities where different approaches can mutually inform and enhance one another. Detailed behavioural analyses of spontaneous interaction can provide insights that experimentation might not have found without the observation of the phenomena in their natural environment in the first place. Such observations and conclusions about mechanisms, however, can often be modelled experimentally or computationally to test hypotheses about causal effects and to shed light on the (neuro)cognitive processes that underpin them. Also, from tests with artificial agents or abstract models, it is essential to go back to the natural human–human interaction to compare behavioural observations, cognitive architectures and neural processes, while with experiments on humans, it is essential to apply paradigms that capture as much of the natural environment and social processes as possible, as laid out above.

In short, to advance the science of face-to-face interaction, we must transgress interdisciplinary boundaries so that the wealth of approaches and techniques we have at our disposal, collectively, provides us with the most comprehensive understanding of face-to-face interaction possible. In an attempt to propel the field forwards, this special issue brings together studies that shed light on the variety of theoretical approaches, methods, tools and foci that result from approaching face-to-face interaction from a multi-disciplinary perspective, and the richness of the insights that can be gained from it. Some of the studies included in this theme issue examine the detail of behaviour on a micro-scale, with smaller samples and precise measurements, while others take a big picture approach to more coarse-grained questions. Some studies focus on verbal behaviour while many others examine the visual components of an interaction. Within these visual behaviours, different studies may examine different modalities—gaze and eye movements, facial movements, hand movements etc. Different participant populations also provide different insights into interaction, ranging from studies of infants and children to adults, and from studies of artificial agents to animals. At present, the wide variety of methods and approaches to understand interaction provides a blossoming of ideas that we hope will lead to a range of important developments.

**Figure 1.**
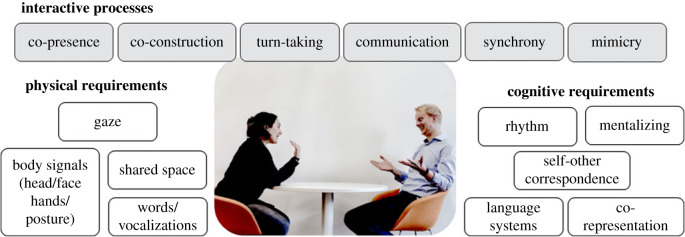
Building blocks of face-to-face interaction. Here we give examples of some interactive processes which cannot be fully studied in solitary individuals; this is not an exhaustive list but summarizes the processes examined in this special issue. For these processes to work, there are particular physical requirements in terms of shared spaces and the use of particular effectors; there are also particular cognitive requirements—processes that have been studied individually but which make a particular contribution or may be engaged differently in interaction.

In figure 1, we summarize some of the interactive processes which this special issue examines. These are processes which cannot be fully studied in isolated individuals, but must be understood in an interactive fashion. This figure also summarizes some of the physical factors which should be present for an interaction to arise. Not all are required for all interactions, but all face-to-face interactions require a bodily mechanism by which individuals can interact as well as a shared physical (or virtual) space. Delineating the physical requirements of interactions may help us categorize and understand them. Interactive processes also have particular cognitive requirements—there may be basic types of cognition which are essential to interaction, and the figure shows some possible examples. Again, delineating which processes contribute to which interactions will be important in understanding if and how interaction builds on simpler cognitive systems.

## 3. Core processes in interaction

Mimicry arises when one person copies the actions of another person and has been seen as a tool for building culture [23] and for strengthening interpersonal bonds [24]. However, the mechanisms by which this can happen and the dynamics of mimicry interactions are less clear. Two papers in the special issue provide new insights. Fabiola and colleagues [25] directly examined how much typical adult participants mimicked the actions of a confederate who was seen face-to-face, on a live video call or in a prerecorded video. Results showed similar mimicry levels between face-to-face and video calls, and both of these resulted in more mimicry than the video condition. This demonstrates one of the basic principles of face-to-face research—that behaviour is different when real people are present (even on a live video call). The results are consistent with previous studies of gaze behaviour in similar conditions [26].

In a second study of mimicry, Hirsch *et al.* [27] developed an innovative paradigm where one participant was able to watch an emotion-inducing movie, while the second participant watched the face of the first. Gaze patterns, facial mimicry and brain activation patterns were recorded from both participants throughout the study, the latter using fNIRS. The results showed that face-watchers would spontaneously imitate the facial expressions of the movie-watchers, and that this was reflected in distinct patterns of brain activity in the face-watchers. Again, this type of study of interactive mimicry could not be implemented in solitary participants and gives new insights into how people may use their faces to communicate emotions with other people.

Visual communication is also important in two papers in the special issue that focus on eye contact. Amici *et al.* [28] provide a detailed examination of patterns of eye contact between mother and infant over the first year of the infant's life, in both zoo chimpanzees and humans living in Germany. They find that mutual gaze durations decrease with age and that there is better gaze coordination in humans. This kind of detailed observation and cataloguing of social behaviour provides an important building block for future theories of the development of gaze and visual communication. In a second developmental study, Kidby *et al.* [29] examine the coordination of gaze and affect between 10-month-old infants and their mothers during solo play and social play. They find that the social play condition leads to more positive affect and more coordination. Interestingly, instances of shared positive affect were more likely to be initiated by the mother, while instances of negative affect were more likely to be initiated by the infant. This links back to the mimicry studies above and demonstrates how mimicry is a two-way interaction even in young infants. Studies of infant behaviour alone (or viewing a computer-controlled stimulus) could not reveal the dynamics of these patterns of behaviour.

The importance of social interaction in the design of artificial agents is also a central theme in a second review paper [30], which focuses on the methods available for designing and evaluating these agents. The review by Gratch describes how interactive agents can be built using a learning-partner approach, in which the responses of participants in a real (or simulated) conversation are used to train a model that will show similar responses. For example, agents can produce back-channel behaviours including mimicry, gaze, smiles and nods in order to act as an ‘active listener’. Such systems can provide an interesting test of theories of social interaction, but the paper warns that they must be built carefully and with due consideration of the types of mentalizing required for the interaction, if they are to provide a valid test of our theories.

Studies of the ability to mentalize—to consider what other people believe or know—have been a cornerstone of research into social cognition in the last four decades. These have led to a detailed understanding of how people interpret movies or picture sequences where others have a false belief [31]. Far fewer studies have examined cases of ‘live mentalizing’, where a participant considers the beliefs/desires of another person who interacts or communicates with them. For example, a movie of another person cannot see or judge the participant, but a second participant (or confederate) who watches or interacts with the participant could also communicate with them or judge them, engaging cognitive processes of mentalizing and reputation management. In this special issue, Freeth & Morgan [32] present a review paper which considers the impact of social presence on mentalizing and other social skills in autistic and non-autistic people. They argue that the social presence of another person has an impact on attention and task performance in neurotypical participants, but it is less clear if this is also true for autistic participants. Measuring and systematically manipulating the potential for social interaction as part of research studies will be important to understand how mentalizing is really used during interactions.

Kuhlen *et al.* [33] also provide a review paper, focused on interactive language production. They focus on language as joint action and review evidence that participants who take turns to name items will represent and show interference effects from their partner's items in the list even if they do not hear their partner say the word. Furthermore, placing classic picture–word interference tasks in the context of a social interaction eliminated interference effects, showing that communication needs can override classic cognitive effects—and thus highlighting how capturing social interaction can critically change cognition. They also review neuroimaging data linking these communication effects to mentalizing regions of the brain.

A very different approach to the challenge of interactive mentalizing is found in the paper from Kahl & Kopp, which reviews the development of artificial agents who can engage in conversation with people [34]. They describe the need for ‘good-enough’ mentalizing systems which can provide a rapid estimate of what another person understands that is sufficient to facilitate conversation, even if it is not perfect. They also advocate for an enactive approach, in which sensorimotor activity is part of cognition and social interaction is essential to social understanding [35]. As an example, interactive agents built using principles of hierarchical predictive processing and good-enough mentalizing are able to learn to communicate in writing until they reach a common understanding. In a second example, an artificial agent can engage in conversations including verbal and non-verbal communication with repair of errors and the use of back-channels to signal understanding. These impressive examples demonstrate the computational principles that are important in creating systems that engage in communication and mentalizing.

Turn-taking is a fundamental building block of social interaction, providing a basic infrastructure for different kinds of conversation from infant proto-conversation to animal calls and adult discussions. Many studies have focused on gaps in conversation turns, building on the observation that in different languages studied around the world turn-taking is characterized by a minimization of gaps and overlaps between turns (with modal turn transition times of between 0 and 200 ms for question-response sequences) [36], leading to questions about what cognitive mechanisms enable this rapid and efficient social coordination. In a detailed motion tracking study, Howes *et al.* [37] examined the timing of turn-taking in groups of three participants which sometimes included one person with schizophrenia. They found longer gaps and less use of gestures for conversational repair in groups which included a person with schizophrenia. In particular, these participants seemed less responsive to conversation cues that might give them a turn, and other participants adapted to this different conversation dynamic.

Turn-taking dynamics were also studied by Templeton *et al.* [38] in a large-scale examination of 261 stranger dyads and 65 friend dyads having short conversations which were later rated for social connectedness. While stranger pairs showed the common effect that short gaps at turn-taking lead to more positive ratings of social connection, this was not true in friend pairs. That is, long conversation pauses between friends were rated positively, possibly because they reflect thoughtful responses to an issue. This important result shows that gap duration may not provide a simple heuristic of conversation quality, and that laboratory studies of friends may give quite different results to studies of strangers.

In a study of the interactions of 5-month-old infants and their mothers, Nguyen *et al.* [39] also examined turn-taking behaviour in conjunction with fNIRS recording of interbrain synchrony. They found that turn-taking behaviour was related to the interpersonal brain synchrony in the interaction, and also to the infant's later vocabulary size at 24 months of age. This shows the importance of early bidirectional communication for later social development. A very different approach to the development of turn-taking can be seen in a study of seal pups from Anichini *et al.* [40]. They studied orphan harbour seals in a care centre, who naturally would call to their mothers in the wild though the mother seals are silent. The orphan seals would vocalize in patterns that depend on their social context (alone or with another seal) and would take care not to call at the same time as another seal. This suggests that fundamental mechanisms for turn-taking might be present even in this species, which might represent the evolutionary basis for processes contributing to turn-taking in humans.

Building on studies of turn-taking, the special issue also includes studies which examine the role of gesture in social coordination. Kendrick *et al.* [41] examined a large corpus of natural conversation to see if gaze and gestures have a role in coordinating turn transitions. They tracked gaze patterns and gesture across dialogues, and find that the presence of gaze-aversion or the presence of an incomplete gesture means that it is less likely that a turn is handed over to the other person. This indicates that turn coordination is not based purely on linguistic features, but also uses visual signals including gaze and gesture in a multi-modal fashion.

The final paper in this special issue considers gesture's role in the evolution of language. Levinson [42] argues for a universal interactional niche at the core of all languages across the world, despite substantial differences in phonemes, syntax and other elements. The paper focuses on spatial concepts as a fundamental feature of language which provide a building block for many grammatical constructions and argues that earlier gestural communication also strongly draws on and communicates spatial concepts (such as spatial gestures that guide and enable effective communication even in people with little common language), thus pointing towards gestural evolutionary roots as the basis of human spoken language.

## 4. Moving forwards

The short summary above of this special issue illustrates the diversity of approaches to the topic of studying social interaction. Starting from the simple building blocks of mimicry, gaze, turns and mentalizing, there are a wide variety of methods and types of question which researchers are actively addressing. One common theme across many of these papers is the importance of studying behaviour as participants are engaged in a genuine interaction, not just a tightly controlled task. Several of the studies provide data or reviews (Freeth [32]; Gratch [30]; Kuhlen [33]; Fabiola [25]) which argue that interactional contexts lead to qualitatively different behaviour compared to more traditional non-interactive contexts.

**Figure 2.**
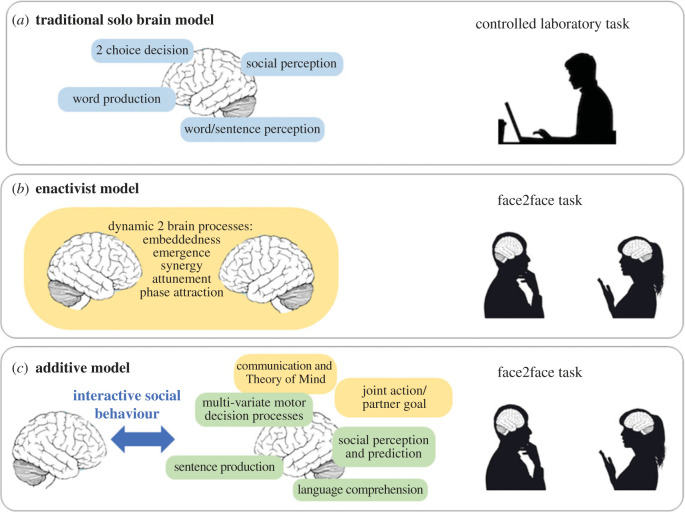
Three approaches to social cognition with different types of theory (left) and experimental methods (right). The traditional solo model (*a*) examines specific tasks in isolated participants. The enactivist approach (*b*) replaces traditional models with a new dynamic model of face-to-face interaction and studies these in dynamic face2face tasks. The additive model (*c*) suggests that solo brain processes may be enhanced and engaged differently in interaction (green boxes) or entirely new processes may be engaged (yellow boxes). These processes are critical in interactive social behaviour which connects brains and can best be studied in face-to-face tasks.

An important question for the future will be—what kind of neurocognitive models should we use to make sense of behaviour in these interactive contexts? To clarify this question, we distinguish three different ways in which researchers might approach the study of social interaction (figure 2). First, the traditionalist might argue that existing methods have served us well and that continued careful experimental designs and laboratory studies will move the field forwards sufficiently. Theories in this area include substantial work on social perception, decision making (typically choosing one of two options based on a particular reward schedule) and the perception and production of single words. Researchers in this traditional framework might in particular argue that the lack of experimental control and repeatability in interactive research studies makes them very hard to interpret, and that such data might provide anecdotes but cannot drive rigorous hypothesis-driven research. Thus, they would prefer well-controlled laboratory tasks where stimuli are pre-defined and participants respond on a computer (figure 2*a*).

In stark contrast, the enactive approach to cognition rejects traditional methods and suggests that there are dynamic cross-brain processes which cannot be captured in traditional single-person studies. Furthermore, this approach claims that it is not possible to understand these processes in terms of single brains, but that concepts of synergy, emergence and attunement that apply across individuals should be central to our models [35]. Such an approach emphasizes the relational and embedded nature of cognitive processes, and suggests that an entirely different type of theory is required to make sense of data from a new type of interactive experiment. This would require a radical overhaul of current methods and theories to create a new and different field of study (figure 2*b*).

The third possibility is an additive approach—one that builds on traditional models but also recognizes that a live social interaction may change cognitive processing and engage additional cognitive processes which are not seen in traditional laboratory studies. For example, processes for social perception engaged when watching a movie might be modulated by context and engaged differently when perceiving live person face-to-face. Other additional cognitive processes might also be engaged in interaction; for example, face-to-face interaction allows the calculation of joint action goals [5] and the engagement of reputation management processes which cannot be seen in solo tasks [1]. The additive model assumes these social processes build on top of the non-social processes typically studied with traditional methods, such as social perception or word processing. Furthermore, these extra cognitive processes required for interaction can still be modelled and understood as processes that happen within a single brain, without the need for an entirely new type of theory [43]. Thus, additive models would use interactive experimental designs, but work to create theories that link closely to the more traditional single-brain framework (figure 2*c*).

The present special issue does not distinguish between these models of how social cognition should be studied and understood, but rather presents new data on different elements of social interaction within these models that represent core building blocks of interaction. These include coordinated behaviours like mimicry and synchrony; rhythm and turn-taking; consideration of other people in terms of mentalizing and co-presence; and visual signalling with facial expressions or gaze or gesture. The contributions focus on the role of these coordinated behaviours in interactions among neurotypical individuals, with individuals from different clinical groups, in child–parent interactions, and among different species. Moreover, they reflect on the past and the future, through their focus on the role of gesture in interaction and language from an evolutionary perspective, as well as the implementation of coordinating behaviours in artificial agents and the virtual environments that will increasingly shape our social interactions in the decades to come.

## 5. Conclusion

As humans, we spend much of our waking hours in face-to-face social interaction on a daily basis, and usually with ease, so much so that it seems trivial at first sight. This special issue draws attention to the incredible complexity of face-to-face social interaction and the multi-level, interdisciplinary approach needed to break new ground and move the science of face-to-face interaction forwards. We advocate the need for bringing different approaches and disciplines together to chart new territory on multiple levels, including the behavioural principles that shape and constitute face-to-face social interactions, the signals by which we achieve coordination, the (neuro)cognitive mechanisms they require as a basis, and the paradigms, tools and methods we need to study them. Recognizing the mutual enrichment that different approaches can offer is critical for this advance, ranging from qualitative analyses giving rise to quantification and experimentation, to experiments on language, cognition and communication based on paradigms that aim to capture social interaction, to neurocognitive studies that move from solo to two (or more) brains in interaction. The idea is not to replace current approaches. They all provide fundamental insights in their own right. Rather, the emphasis is on adding to what we can learn from extant approaches by bringing them together, thus helping us to refine our insights and adding further pieces to the complex puzzle of human face-to-face interaction.

## Data Availability

This article has no additional data.
